# An Expanded Platform for Young Scientists

**DOI:** 10.1128/mSphere.00653-19

**Published:** 2019-09-18

**Authors:** Michael J. Imperiale, Ira J. Blader

**Affiliations:** a University of Michigan Medical School, Ann Arbor, Michigan, USA; b University at Buffalo, Buffalo, New York, USA

## EDITORIAL

Earlier this year, *mSphere* began publishing commentaries from young microbiologists in which we asked them to talk about a manuscript or two that strongly influenced the way that they think about their science. These commentaries, which we call mSphere of Influence (mSI), have been thoughtful and thought provoking, and the response has been tremendous. First, there has been incredible enthusiasm among this generation of scientists to contribute their stories. Second, we have been notifying the authors of the papers to which the commentaries refer, and those authors have been thrilled to see their work cited in this manner.

One of the mSI articles that we published, from Megan Spurgeon at the University of Wisconsin—Madison, took a slightly different tack (1). Rather than writing about her exciting research on human papillomavirus, Megan talked about how she was influenced to think about the scientific enterprise more generally and how we all have important roles to play in it.

Today, we are posting two more mSI articles that are off the beaten path, so to speak. Both of these come from junior faculty who are also members of ethnic groups that are underrepresented in scientific research: Kat Milligan-Myhre from the University of Alaska, Anchorage, and Michael Johnson from the University of Arizona. In their articles (2, 3), they talk about some of the challenges they see as underrepresented minorities (URMs).

Kat, who is a native Alaskan Inupiat, writes about how she has dealt with the cultural differences that she has experienced during her professional journey, concluding that these differences are actually strengths that she has embraced as she has started her faculty work at the University of Alaska (2).

Michael is an African-American scientist who writes about the challenges that URM scientists face in competing for faculty positions (3). He notes the discrepancy between the composition of the candidate pool and the actual composition of the junior faculty ranks and implores university leadership to increase their efforts to close the gap.

As editors of a journal with high visibility in the field of microbial sciences, we feel that it is important that these stories be told. First, we strongly believe that URM scientists need to be heard and listened to. Second, we understand that for our field to thrive, we must value the contributions of as diverse a group of scientists as possible.

At *mSphere*, we have tried to incorporate diversity at all levels. We are proud that we have achieved gender equality among our Senior Editors and Editors. On the other hand, we are not as well balanced with respect to other demographics. We cannot correct these discrepancies overnight, but we are committed to continue to pay heed to these issues and work toward greater equality. We welcome your comments as we continue to improve at *mSphere*!
